# Anti-Retroviral Therapy Is Associated with Decreased Alveolar Glutathione Levels Even in Healthy HIV-Infected Individuals

**DOI:** 10.1371/journal.pone.0088630

**Published:** 2014-02-12

**Authors:** Sushma K. Cribbs, David M. Guidot, Greg S. Martin, Jeffrey Lennox, Lou Ann Brown

**Affiliations:** 1 Department of Medicine, Division of Pulmonary, Allergy and Critical Care, Emory University, Atlanta, Georgia, United States of America; 2 Department of Medicine, Division of Infectious Diseases, Emory University, Atlanta, Georgia, United States of America; 3 Department of Pediatrics, Division of Neonatal-Perinatal Medicine, Emory University, Atlanta, Georgia, United States of America; University of Puerto Rico, Medical Sciences Campus, Puerto Rico

## Abstract

**Objective:**

Lung infections are a leading cause of death in HIV-infected individuals. Measuring redox in HIV-infected individuals may identify those with chronic oxidative stress who are at increased risk for lung infection. We sought to estimate the association between HIV infection and oxidative stress in the lung, as reflected by decreased levels of glutathione and cysteine in the epithelial lining fluid.

**Methods:**

Bronchoalveolar lavage (BAL) fluid was collected from healthy HIV-infected subjects and controls. Individuals were excluded if they had evidence of major medical co-morbidities, were malnourished or smoked cigarettes.

**Results:**

We enrolled 22 otherwise healthy HIV and 21 non-HIV subjects. Among the HIV-infected subjects, 72.7% were on anti-retroviral therapy (ART) with a median CD4 count of 438 (279.8–599) and viral load of 0 (0–1.0) log copies/mL. There were no significant differences in median BAL fluid glutathione and cysteine levels between HIV and HIV-uninfected subjects. However, BAL glutathione was significantly higher in HIV-infected subjects on anti-retroviral therapy (ART) compared to those not on ART [367.4 (102–965.3) nM vs. 30.8 (1.0–112.1) nM, p = 0.008]. Further, HIV infection with ART was associated with an OR of 2.02 for increased BAL glutathione when adjusted for age and body mass index, whereas HIV infection without ART was associated with an OR of 2.17 for decreased BAL glutathione.

**Conclusion:**

HIV infection without ART was associated with increased oxidative stress, as reflected by decreased alveolar glutathione levels, in otherwise healthy HIV-infected individuals. Further study needs to be done identify predictors of lung health in HIV and to address the role of ART in improving lung immunity.

## Introduction

Human immunodeficiency virus (HIV) infection remains an important problem worldwide. The Centers for Disease Control (CDC) estimates that 1,148,200 people older than 13 years of age are living with HIV infection in the United States, including 207,600 who are unaware they are infected [1]. Before the widespread use of combination anti-retroviral therapy (ART), pulmonary diseases were among the most common complications of HIV infection and were frequently associated with significant morbidity and mortality [2], [3]. Although ART has substantially decreased opportunistic infections associated with HIV infection, pneumonias from these ‘routine’ pathogens such as pneumococcus and influenza continue to cause significant morbidity and mortality in these susceptible individuals [4], [5]. Advanced immunosuppression is a known risk factor and even single episode of pneumonia is associated with increased morbidity and mortality [6].

It is unclear at this time the exact mechanism by which HIV infection renders individuals susceptible to lung infections. One possibility is the generation of reactive oxygen species, resulting in increased oxidative stress within the alveolar space. The lungs are constantly exposed to oxidants, both internally and from the environment. The ability to neutralize these oxidants is essential for lung health [7]. Oxidative stress can be generated from a number of different mechanisms [8]. The relative oxidation of extracellular thiol disulfide pairs such as cysteine/cystine (Cys/CySS) and glutathione/glutathione disulfide (GSH/GSSG) determines the redox potential in a biological microenvironment [7]–[9]. Glutathione (GSH) is considered the primary antioxidant in the alveolar space and the GSH/GSSG and Cys/CySS redox couples comprise the major low-molecular weight thiol/disulfide redox control systems in mammals [10]. Its concentration is more than 20-fold that in plasma and over 90% is in the reduced form. GSH is able to scavenge reactive oxygen species and provides the principal intracellular defense against oxidative stress. Cys is a rate-limiting component of GSH synthesis and plays an important role in maintaining the detoxification of free radicals and reactive oxygen species.

Chronic HIV infection alone has been known to cause significant oxidative stress systemically, both in pre-clinical and clinical studies, and both GSH and Cys have been extensively studied in this population. GSH levels were found to be decreased greater than 90% in HIV transgenic rats compared to wild-type and GSSG:GSH ratios were increased 3-fold [11]. Post-lipopolysaccharide treatment, HIV-infected animals also have decreased GSH, increased nitric oxide metabolites and superoxide [12]. Clinically, reduced plasma Cys was significantly lower in HIV-positive patients compared to control subjects [13] and several studies have shown that HIV-infected patients have disturbances in GSH redox balance[14]. GSH deficiency has even been associated with impaired survival in HIV-infected subjects [15]. In the lung, chronic HIV transgene expression in animal models causes significant alveolar oxidative stress [12]. In clinical studies, the results have been conflicting. Pacht et al. reported that the concentration of GSH in the epithelial lining fluid was similar between HIV and non-HIV subjects [16]; however, these same authors demonstrated that GSH levels in the epithelial lining fluid were found to be significantly decreased over time [17]. Others have also shown that HIV-infected subjects had a deficiency of GSH in the lung [18], [19]. The effect of ART on oxidative stress biomarkers is still controversial. Awodele et al. found that HIV-infected subjects without ART had lower systemic GSH levels and higher lipid peroxidation as compared to HIV-infected subjects on ART [20]. However, the effect of anti-retroviral therapy (ART) on oxidative stress, as measured by GSH and Cys levels, in the lung has not been previously studied.

We sought to estimate the association between HIV infection and oxidative stress in the lung, as reflected by decreased levels of GSH and Cys in the epithelial lining fluid. We enrolled a cohort of non-smoking, otherwise healthy HIV-infected individuals on and off ART to examine this effect. We hypothesized that HIV infection would be associated with increased oxidative stress in the lung, as reflected by decreased GSH and Cys levels in the epithelial lining fluid.

## Methods

### Study design and protocol

All participants provided written informed consent to participate in the study. The research study was discussed with the subject. The study purpose, procedures, risks and benefits, and alternative treatment options were described in detail. The subject was given opportunity to read the informed consent form and ask questions. The subject also verbalized understanding of the study and all related visits and procedures, verbalized understanding of the HIPAA authorization, signed and received a copy of the informed consent form. No study procedures were performed prior to obtaining informed consent. This research study, including the consent procedure, was approved by the Institutional Review Board at Emory University and the Grady Research Oversight Committee. We performed a cross-sectional study of HIV-infected subjects without other medical problems and compared them to healthy (HIV-uninfected) subjects within the Grady Health System in Atlanta, GA. This study was approved by the Emory Institutional Review Board and the Grady Research Oversight Committee. Informed consent was obtained from all subjects. Inclusion criteria included all subjects with and without HIV infection. Exclusion criteria included active liver disease (known cirrhosis and/or total bilirubin >2.0 mg/dL), heart disease (ejection fraction <50%, history of acute myocardial infarction, New York Heart Association (NYHA) II-IV cardiac symptoms, severe valvular dysfunction), current renal disease (dialysis dependent or creatinine >2.0 mg/dL), current lung disease (spirometry revealing a forced vital capacity (FVC) or forced expiratory volume in 1 second (FEV1) <80% of predicted), diabetes, current pregnancy, malnutrition (body mass index <17), current tobacco use and age <25 years.

The study coordinator entered the date, patient's name, medical record number, and study ID number in the patient enrollment log. Data were initially collected using our established case report forms and then entered into a HIPAA-compliant, secure database. All subjects completed a pre-enrollment evaluation (visit 1) which included: 1) complete history and physical exam, 2) routine blood chemistries (basic chemistry, liver function tests, complete blood count, coagulation parameters, hemoglobin A1C), 3) CD4+ count and viral load (if not done in past 30 days), 4) urine pregnancy test (qualitative beta-HCG) for women of child-bearing potential, 5) urine dipstick for cotinine, 6) spirometry (FEV1, FVC), 7) Short Michigan Alcohol Screening Test (SMAST) and Alcohol Use Disorders Identification Test (AUDIT) alcohol use questionnaires, and 8) Body mass index (BMI). Demographic data were collected. Subjects with exclusions identified at the time of the pre-enrollment evaluation were excluded from further participation.

After completing the pre-enrollment evaluation to confirm eligibility, subjects presented to the Grady Memorial Hospital Clinical Interaction Site after an overnight fast. Enrolled volunteers had the following interventions on visit 2∶1) CD4+ count and viral load for HIV-infected subjects only and 2) Flexible fiberoptic bronchoscopy with standardized bronchoalveolar lavage (BAL) performed using standard conscious sedation techniques. BAL was performed by installing 30 mL aliquots of 0.9% non-bacteriostatic normal saline solution, followed by withdrawal with low-pressure hand suction until a total of 180 mL had been administered. Subjects were contacted by phone 24 hours after completing the study to ensure patient safety. A Data Safety Monitoring Representative was appointed to review each patient's enrollment procedures and bronchoscopy report on a monthly basis.

BAL fluid was transported immediately to a research lab at Emory University. All samples were de-identified without any patient identifiers. In addition, lab personnel were unaware as to whether the BAL sample was from a HIV-infected subject or a control subject. BAL samples were preserved immediately after collection in 5% perchloric acid solution containing iodoacetic acid (6.7 µM) and boric acid (0.1 M). This step was completed within 30 min of collection to prevent degradation or oxidation of GSH. After protein removal, samples were derivatized with dansyl chloride and separated on a 10-µm Ultrasil amino column by high performance liquid chromatography (HPLC) (Waters 2690; Waters Corp., Milford MA). Fluorescence detection was recorded by two detectors. This procedure has been done before by this lab [21]. GSH, GSSG, Cys, and CySS were quantified relative to α-glutamyl-glutamate, an internal standard.

### Sample size calculations

The primary outcome variables included GSH and Cys levels in the BAL. The primary variable of interest was HIV infection. Potential confounders included age, BMI, and use of ART because they have been shown to be associated with the outcome and HIV infection in previous studies. In our cohort of HIV and HIV-uninfected subjects, we conducted a study with GSH and Cys, two continuous response variables from independent HIV and non-HIV subjects with 1 non-HIV per HIV subject. Consistent with the results of a previous study [22], we assume that the response within each subject group for BAL GSH will be within a standard deviation of 2754.9 nM. If the true difference in the HIV and non-HIV means is 6200 nM, we need to study 5 HIV-infected subjects and 5 HIV-uninfected subjects to be able to reject the null hypothesis that the population means of the HIV and non-HIV groups are equal with probability of 0.9 at a significance level of 0.05.

### Statistical Analyses

Univariate comparisons between HIV subjects and non-HIV subjects were calculated and evaluated for a significance level of 0.05 using a chi-squared test for categorical variables and a two-sample t-test for continuous variables. The data was log-transformed or a Wilcoxon Rank-Sum Test was used when the data was not normally distributed. To examine BAL GSH and CYS levels among those with and without HIV, multi-variable linear regression analysis was used with log-transformed BAL GSH and BAL Cys levels as the outcome controlling for age, BMI and use of ART. Three exposure groups were analyzed – those with HIV on ART, those with HIV without ART and those without HIV. All analyses were conducted using NCSS or SAS software.

## Results

### Baseline Characteristics of HIV-infected Subjects and HIV-uninfected Subjects

A total of 22 HIV and 21 non-HIV subjects were enrolled. 17 HIV and 19 non-HIV subjects had BAL oxidative stress markers. **Table 1** shows the demographic characteristics of HIV and HIV-uninfected subjects enrolled in the study. The mean age of the HIV-infected subjects was significantly greater than the mean age of the HIV-uninfected subjects (47.8 years +/− 7.0 vs. 40.5 years +/− 10.5, p = 0.01). The majority of subjects, both HIV and HIV-uninfected, were African American. There were no statistically significant differences in gender or body mass index (BMI) between the groups.

**Table 1:** Demographic characteristics of HIV-infected Subjects and Non-HIV-infected Subjects Enrolled in the Study.

Patient Characteristics	Non-HIV N = 21	HIV N = 22	*P*-value
**Age (mean, SD)**	40.5 (10.5)	47.8 (7.0)	0.01
**Gender (% male)**	9 (42.8)	9 (40.9)	0.89
**Race**			0.33
** White n (%)**	5 (23.8)	2 (9.1)	
** Black n (%)**	14 (66.7)	20 (90.9)	
** Other n (%)**	2 (9.5)	0	
**BMI (kg/m^2^, mean, SD)**	31.0 (7.9)	34.3 (8.0)	0.17
**SMAST (median, IQR)**	0 (0-0)	0 (0–3)	0.12
**AUDIT (median, IQR)**	1.0 (0–2.5)	1.5 (0–2.5)	0.64
**CD4 (n = 16) (median, IQR)**		438 (279.8–599)	
**Viral load (log copies/mL, median, IQR)**		0 (0–1.0)	
**Use of ART (%)**		72.7%	

BMI =  Body Mass Index

SMAST =  Short Michigan Alcohol Screening Test.

AUDIT =  Alcohol Use Disorders Identification Test.

ART =  Anti-retroviral medications.

### Baseline Characteristics of HIV-Infected Subjects Only

Among all the HIV-infected subjects, the median CD4 count was 438 (IQR 279.8–599) with a median viral load of 0 (IQR 0–1.0) (**Table 2**). 72.7% of the HIV-infected subjects were on ART. 27.3% of subjects were not on ART due to the following reasons: CD4>500 cells/mm3, unable to tolerate side effects, or non-compliance with the medications. There were no statistically significant differences in age, gender, race, BMI, or alcohol use between those HIV-infected subjects on and off ART. Subjects without ART had a greater CD4 count than those subjects on ART [541.5 (IQR 342–618) vs. 405.5 (IQR 269.3–599), p = 0.54]. In addition, those HIV-infected subjects without ART had a statistically significant higher viral load than HIV-infected subjects on ART [4.08 (IQR 0–4.18) vs. 0 (IQR 0–0), p = 0.04].

**Table 2:** Demographic characteristics of HIV-infected Subjects Enrolled in the Study.

Patient Characteristics	ART N = 16	No ART N = 6	*P*-value
**Age (mean, SD)**	48.4 (5.6)	46 (10.4)	0.49
**Gender (% male)**	6 (37.5)	3 (50)	0.60
**Race**			0.45
** White n (%)**	1 (6.3)	1 (16.7)	
** Black n (%)**	15 (93.7)	5 (83.3)	
** Other n (%)**	0	0	
**BMI (kg/m^2^, mean, SD)**	33.6 (8.4)	36.3 (6.9)	0.48
**SMAST (median, IQR)**	0 (0–3.75)	0 (0–2.3)	0.46
**AUDIT (median, IQR)**	0.5 (0–3.5)	2 (0.8–2.5)	0.46
**CD4 (n = 16)(median, IQR)**	405.5 (269.3–599)	541.5 (342–618)	0.54
**Viral load (log copies/mL, median, IQR)**	0 (0–0)	4.08 (0–4.18)	0.04

BMI =  Body Mass Index.

SMAST =  Short Michigan Alcohol Screening Test.

AUDIT =  Alcohol Use Disorders Identification Test.

ART =  Anti-retroviral medications.

### BAL Glutathione and Cysteine Levels in HIV versus HIV-uninfected Subjects

**Figure 1:**
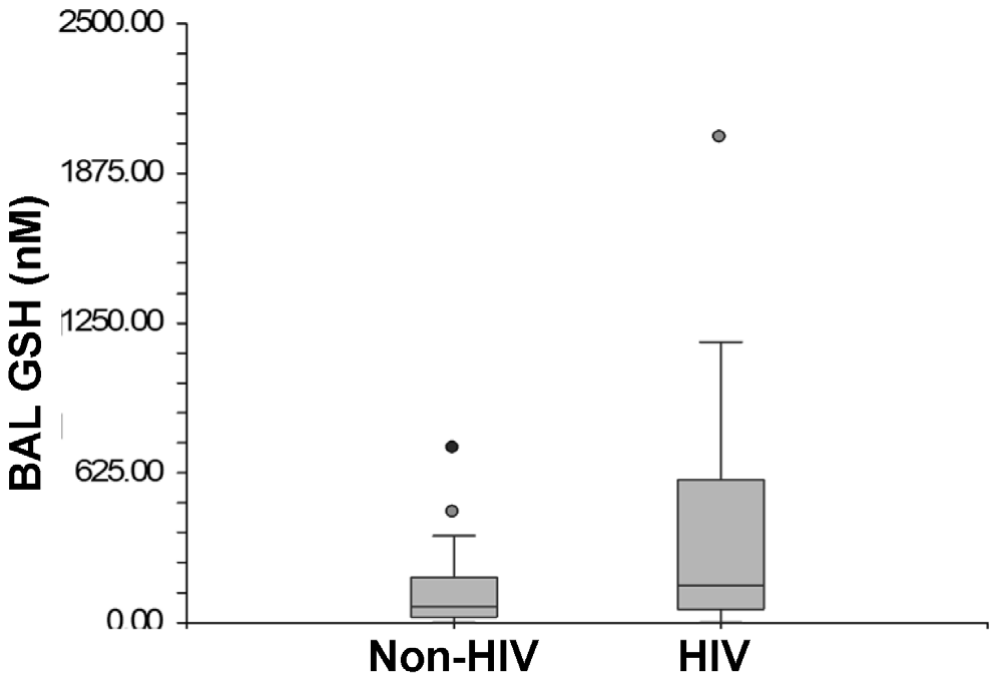
Box Plots of Glutathione Levels in Bronchoalveolar Lavage between non-HIV and HIV-infected Subjects.

**Figure 2:**
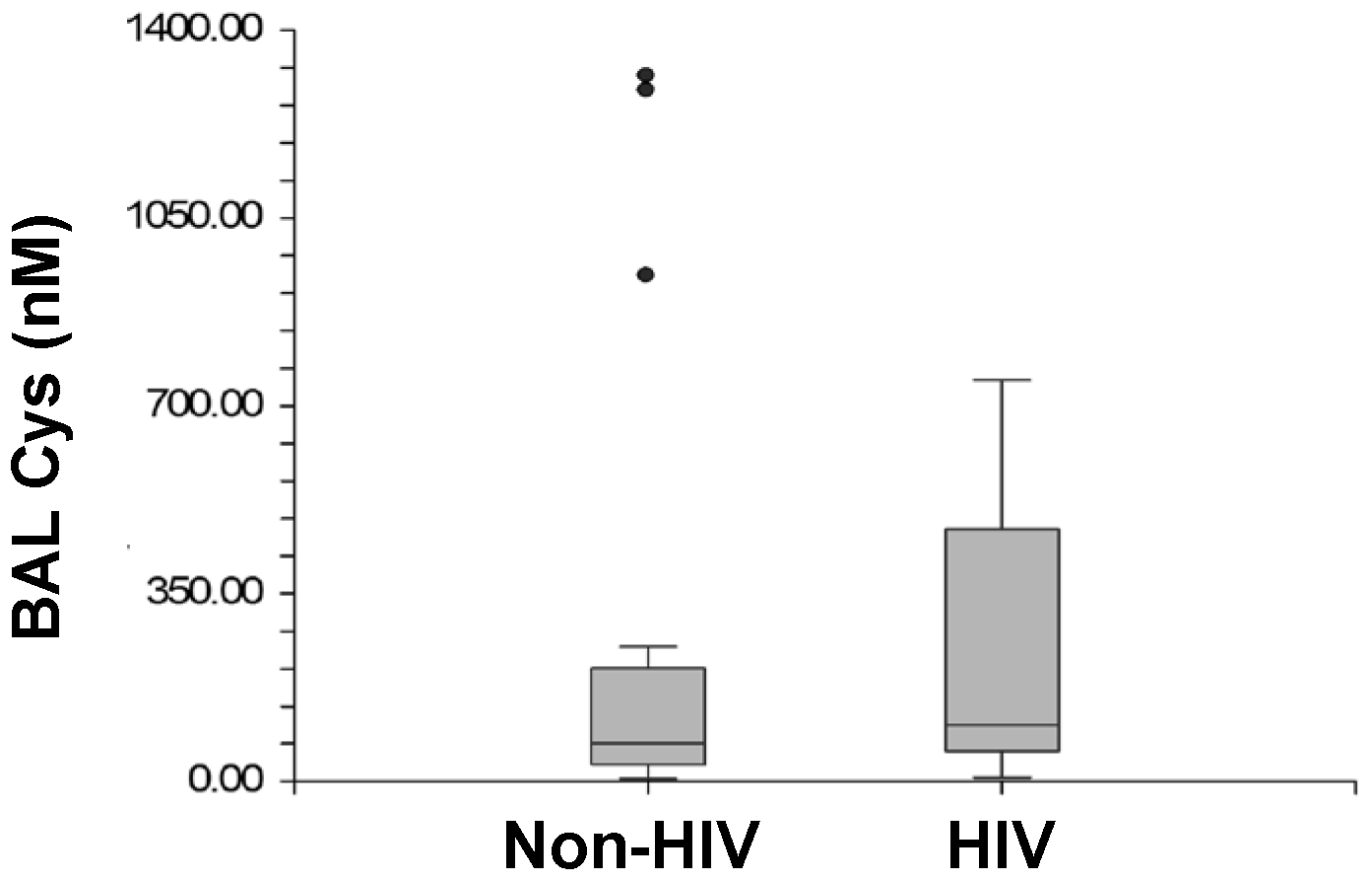
Box Plots of Cysteine Levels in Bronchoalveolar Lavage between non-HIV and HIV-infected Subjects.

There was no statistical difference detected in median BAL fluid GSH levels between HIV-infected and HIV-uninfected subjects [163.7 nM (IQR 55.7–595.9) vs. 71.1 nM (IQR 24.0–188.0), respectively, p = 0.06] (**Figure 1**). There were also no differences in median BAL fluid Cys levels between HIV-infected and HIV-uninfected subjects [108.6 nM (IQR 56.7–470.1) vs. 75.0 nM (IQR 30.8–211.1) respectively, p = 0.40] (**Figure 2**).

### Effect of Anti-retroviral Therapy on Glutathione and Cysteine BAL levels

**Figure 3:**
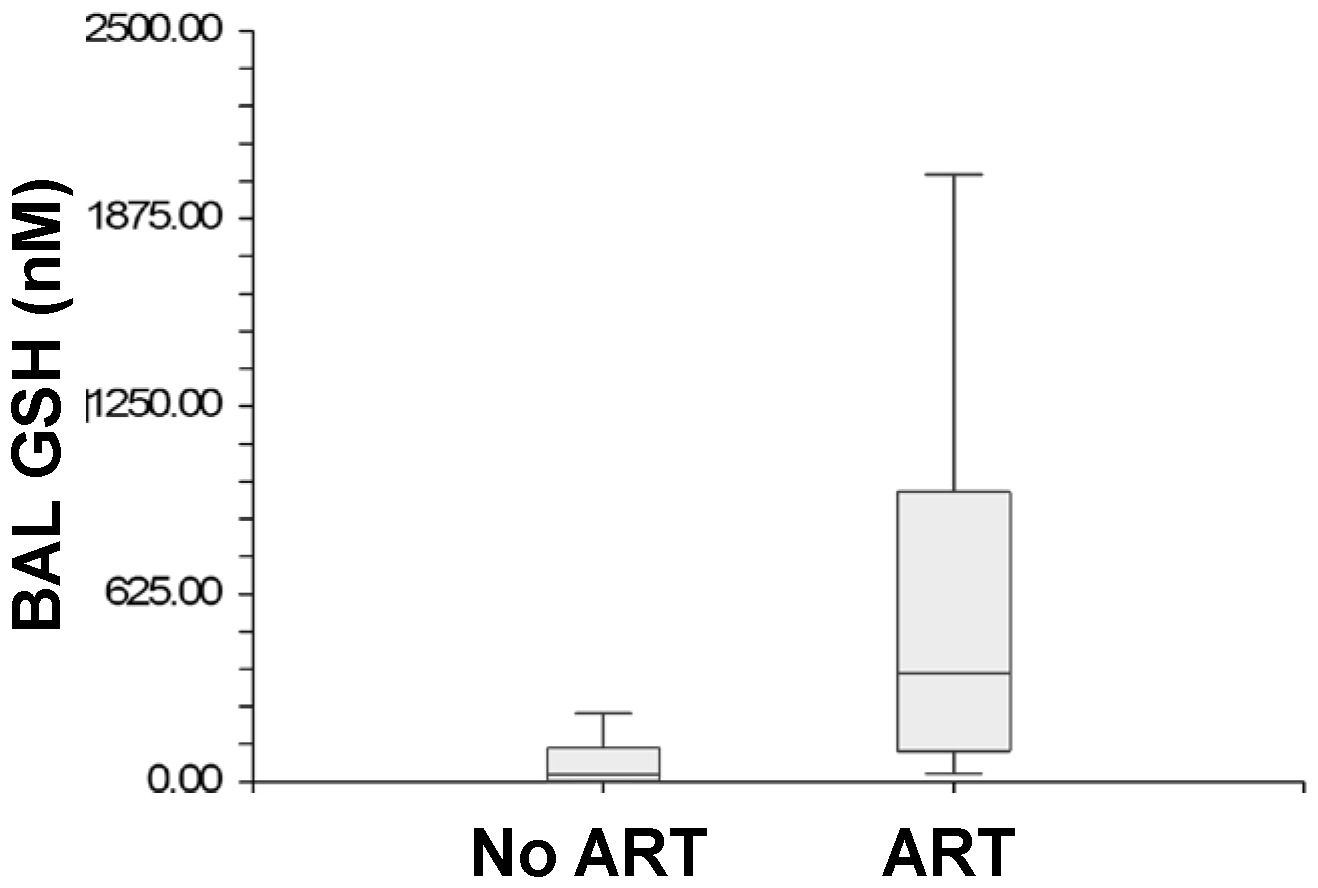
Box Plots of Glutathione Levels in Bronchoalveolar Lavage HIV-infected Subjects on and off Anti-retroviral Therapy.

**Figure 4:**
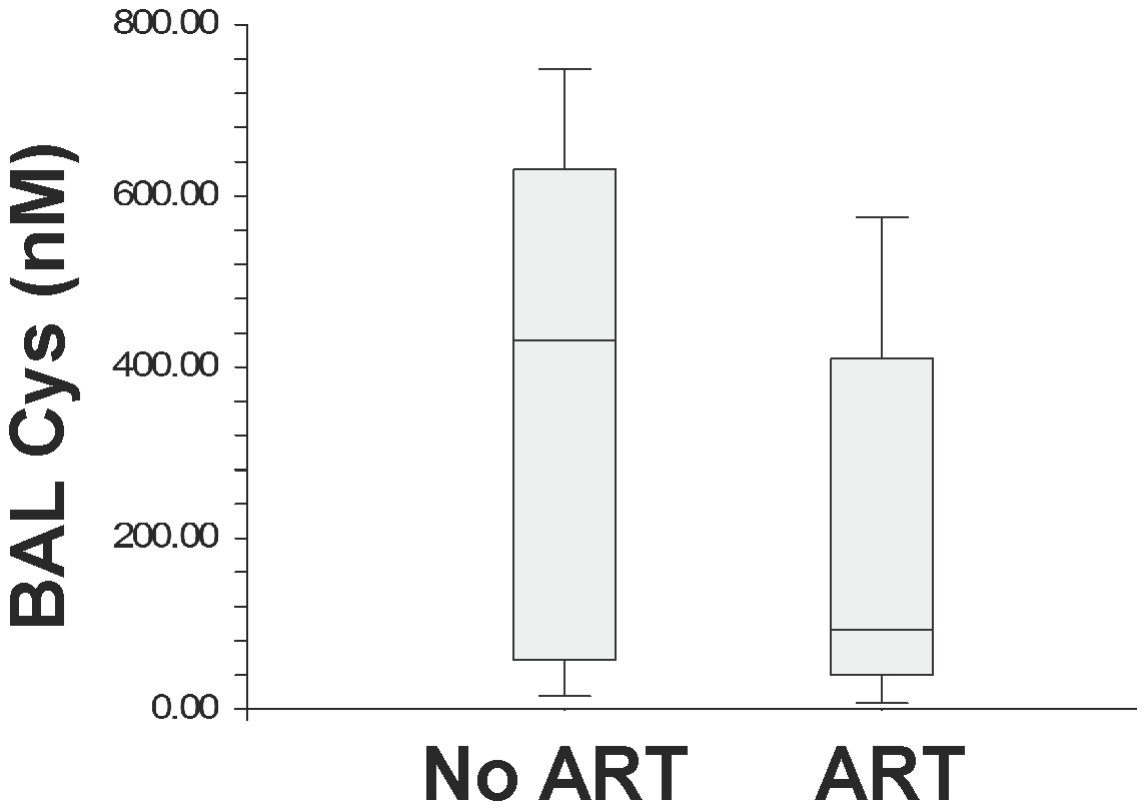
Box Plots of Cysteine Levels in Bronchoalveolar Lavage HIV-infected Subjects on and off Anti-retroviral Therapy.

GSH and Cys levels were also evaluated among only HIV-infected subjects on and off ART. BAL fluid GSH was significantly higher in HIV-infected subjects on ART compared to those without ART [367.4 (IQR 102–965.3) vs. 30.8 (IQR 1–112.1), p = 0.008] (**Figure 3**). Although not statistically significant, BAL fluid Cys was higher in HIV-infected subjects without ART compared to those on ART [433.1 (IQR 57.7–630) vs. 95.7 (IQR 40.2–409.5), p = 0.14] (**Figure 4**). The association of HIV infection with BAL fluid GSH and Cys levels was determined using multiple linear regression with age, BMI and use of ART entered as potential confounders into the model. HIV infection without ART was associated with an estimated OR of 2.17 for decreased BAL GSH. Meanwhile, HIV infection with ART was associated with an estimated OR of 2.02 for increased BAL GSH, (**Table 3**). There was no association observed between BAL Cys, HIV infection, age, BMI and use of ART (**Table 4**). Also, there was no interaction detected between predictor variables in either model.

**Table 3:** Multiple Linear Regression Model for BAL Glutathione.

Variable	Slope	SE	p-value
HIV on ART (vs. no HIV)	0.7032	0.2635	0.01
HIV not on ART (vs. no HIV)	−0.7743	0.3518	0.03
Age (per year)	−0.0051	0.0129	0.70
BMI (kg/m^2^)	0.0126	0.0146	0.39

p = 0.003.

Adjusted R^2^ = 0.27.

**Table 4:** Multiple Linear Regression Model for BAL Cysteine.

Variable	Slope	SE	p-value
HIV on ART (vs. no HIV)	0.0356	0.2299	0.16
HIV not on ART (vs. no HIV)	0.3799	0.3070	0.26
Age (per year)	−0.0034	0.0113	0.64
BMI (kg/m^2^)	0.0029	0.0127	0.99

P = 0.76.

Adjusted R^2^ = −0.05.

## Discussion

The results from this cross-sectional study show that HIV-infected subjects without ART had significantly increased oxidative stress in the lung, as reflected by low BAL GSH levels, compared to HIV-infected subjects on ART. Similar results were seen after adjusting for age and BMI. These results suggest that the use of ART protected the lung from oxidative stress, as reflected by decreased levels of GSH. These associations were not seen with BAL Cys. Although several studies have looked at various systemic oxidative stress biomarkers in HIV-infected individuals on ART [23], this is the first study to evaluate the impact of ART on alveolar oxidative stress markers in otherwise healthy HIV-infected individuals. There were no significant differences in BAL GSH or Cys levels between HIV and HIV-uninfected subjects as a whole.

GSH is the most abundant non-protein thiol in living organisms and essential for many biologic functions including cell defense, T and B cell differentiation, and cytotoxic T-cell activation[24]. It also functions as an antioxidant, scavenging reactive oxygen species and reducing hydrogen peroxide. Not just systemically, but GSH is also considered a primary antioxidant in the lung [25] and the alveolar space. Several studies have shown that HIV-infected subjects have disturbances in GSH redox balance [14] and HIV-associated disturbances in GSH have even been associated with impaired survival [15]. Alveolar GSH is likely to be very important in HIV-infected individuals as well. Alveolar macrophages, the primary host immune defense cells in the lung, have been shown to release exaggerated amounts of superoxide anion even in asymptomatic HIV-infected individuals [26]. Previous investigations have shown that GSH protects alveolar macrophages from hyperoxia-induced injury and injury to Type II alveolar epithelial cells [27]. Therefore, GSH may serve as a critical antioxidant in this environment of oxidative stress.

Although ART has resulted in dramatic clinical and immunological improvements in HIV-infected patients, not all individuals experience a favorable response [28]. Few studies have examined the effect of ART on oxidative stress parameters and these studies remain conflicting. Aukrust and colleagues found that not only did ART decrease viral load and increase CD4+ T cell count, but there was an improvement in the abnormal GSH redox status [14] as well. Others have shown that ART is associated with increased oxidative stress [29]. However, both studies measured plasma biomarkers of oxidative stress and not biomarkers in the alveolar space. In this study, we demonstrated that HIV infection without ART was associated with lower GSH levels in the lung as opposed to HIV infection with ART, suggesting that ART may be protective in the lung even in otherwise healthy HIV-infected individuals. However, although ART has been shown to improve oxidative stress, previous studies have shown that ART did not fully normalize the abnormal redox status [14]. This suggests that a therapeutic intervention aimed at reducing oxidative stress may be beneficial to improve lung immunity, even for those individuals on ART.

There are several limitations with this study. Given that this is an observational cross-sectional study it is subject to confounding by known and unknown measures. However, we did attempt to minimize this by restricting our study population and adjusting for known confounders such as age in our model. Also, approximately 27.3% of the HIV-infected subjects were not on ART for varied reasons; this translates into 6 subjects which is a small sample size and one that may reflect sampling bias. Some of the subjects had CD4 counts greater than 500 cells/mm, whereas others were simply non-compliant with ART. This could have influenced our results; however, we were able to analyze three exposure groups – those with HIV on ART, those with HIV without ART and those without HIV allowing us to assess the effect of ART on alveolar oxidative stress markers which has not been done before.

In conclusion, our study demonstrates that HIV infection with ART was associated with lower oxidative stress in the lung, as reflected by lower BAL GSH levels, suggesting that ART in this population of otherwise healthy HIV-infected individuals is protective in the alveolar space and may improve lung immunity. However, previous studies have shown that ART does not completely restore the disruption in redox pathways. Therefore, further study needs to be done to address the role of antioxidants, particularly GSH supplementation to mitigate HIV-induced oxidative stress and enhance lung health in HIV-infected individuals.
